# Association between Human Leukocyte Antigen (HLA) DQB1*06 and HLA DQB1*03 and adverse outcomes in a group of critically ill patients with COVID-19 in Tunisia: a cross-sectional study

**DOI:** 10.11604/pamj.2023.45.109.39956

**Published:** 2023-06-27

**Authors:** Amène Ben Bnina, Yasmine El Bahri, Amény Cheybi, Nada Ben Lazrek, Syrine Chouchane, Asma Omezzine, Walid Naija, Amina Bouatay

**Affiliations:** 1 Hematology Laboratory, Sahloul Teaching Hospital, Sousse, Tunisia,; 2 Faculty of Pharmacy, University of Monastir, Monastir, Tunisia,; 3 Faculty of Medicine, University of Sousse, Sousse, Tunisia,; 4 Department of Anesthesia and Intensive Care, Sahloul Teaching Hospital, Sousse, Tunisia,; 5 Biochemistry Laboratory, Sahloul Teaching Hospital, Sousse, Tunisia

**Keywords:** Human leukocyte antigen (HLA), COVID-19, critically ill, prognosis, mortality

## Abstract

**Introduction:**

Human Leukocyte Antigen (HLA) system is a highly polymorphic genetic system associated with the prognosis of several infectious diseases. The aim of this study is to investigate the association of HLA polymorphism with the outcome of coronavirus disease 2019 (COVID-19) in Tunisian critically ill patients.

**Methods:**

this retrospective cross-sectional study included 42 consecutive patients hospitalized in intensive care unit (ICU) for COVID-19 in March 2021. Genotyping of HLA loci was performed by LABType™ sequence-specific oligonucleotide (SSO) typing kits (One lambda Inc, USA). Statistical analyses were performed using Statistical Package for Social Sciences (SPSS®) version 23.0. A p-value <0.05 was considered significant. Multivariable regression analysis was performed for the association between HLA polymorphism with adverse outcomes with adjustment for potential confounders such as age, sex, co-morbidities and blood type.

**Results:**

patients included in our study had a mean age of 64.5 ± 11.5 (34-83) years and were mainly men (64.3%; (n=27)). The most common cardiovascular risk factors were obesity (61.9%; (n=26)) and hypertension (26.2%; (n=11)). Thirty-two patients died (76.2%). Eleven patients (26.2%) required intubation during hospitalization. We found that HLA DQB1*06 allele was significantly associated with protection against mortality aOR: 0.066, 95% CI 0.005-0.821; p = 0.035. HLA DQB1*03 allele was significantly associated with protection against intubation aOR: 0.151, 95% CI 0.023-0.976; p = 0.047.

**Conclusion:**

it was found that there are 2 protective HLA alleles against COVID-19 severity and mortality in critically ill patients. This could allow focusing on people genetically predisposed to develop severe forms of COVID-19.

## Introduction

The clinical manifestations of coronavirus disease 2019 (COVID-19) are highly variable in patients. This severe acute respiratory syndrome coronavirus 2 (SARS-CoV-2) infection ranges from a simple asymptomatic form or mild flu-like symptoms to a severe or critical form leading to hospitalization in an intensive care unit (ICU) [1-4].

Several factors can explain this inter-individual clinical variability of COVID-19, namely age, gender, and specific comorbidities of each patient [3]. However, it has been found that patients with an apparently common personal and medical history have extremely variable clinical forms of COVID-19 [3,4]. Thus, other factors may be involved in the pathogenesis of COVID-19, namely genetic factors. The study of the variability of the genetic basis of anti-viral immune response could also explain the inter-individual variability of COVID-19 symptoms [5,6].

Human Leukocyte Antigen (HLA) system is a very polymorphic genetic system that plays a crucial role in anti-viral immune response. HLA antigens present a multitude of viral epitopes to T cells and are involved in the initiation of an immune response. Allelic polymorphism of the HLA system can be associated with the predisposition, protection, or the course of several infectious diseases [6,7]. In the same context, the association of HLA polymorphism with the course and severity of COVID-19 has been evoked by several authors [8-14]. However, the results and conclusions of these studies were different. Furthermore, to our knowledge, an association of HLA polymorphism with COVID-19 is still not known in Tunisian patients. Thus, the objective of this work was to search for an association between HLA antigens and the evolution of COVID-19 in Tunisian patients hospitalized in ICU for SARS-CoV-2 infection.

## Methods

**Study design and setting:** this retrospective cross-sectional study was conducted to investigate the association between HLA polymorphism and COVID-19 outcome in ICU hospitalized patients. The study was conducted in Sahloul University Hospital in March 2021. Sahloul University Hospital is a 683-bed hospital located in the city of Sousse, a coastal city in Central Tunisia. This hospital drains patients mainly from the governorate of Sousse, Kairouan, Kasserine and Sidi Bouzid.

**Study population:** this study involved 42 patients hospitalized in ICU for COVID-19. Included patients were all consecutive patients older than 18 years, hospitalized in ICU for severe COVID-19, and were not vaccinated against SARS-CoV-2. Our exclusion criteria were pregnancy, active cancer, and incomplete data in medical files.

**Data collection:** demographic data (age, gender, and comorbidities), clinical and biological findings (blood type) were collected either by consulting medical files or by referring to electronic hospital medical records. Data collection was completed in 3 weeks.

**Study variables:** variables included were HLA alleles, age, gender, smoking, asthma, comorbidities, symptoms, blood group, intubation and death.

**Definitions:** SARS-CoV-2 infection was confirmed by detection of virus´ genome in nasopharyngeal swab samples by real time reverse transcriptase-polymerase chain reaction (RT-PCR). Intubation is the catheterization of the trachea through the glottis, by a probe introduced through the mouth or nose. Its purpose is to protect the airways, in case of disturbance of consciousness, ventilatory or hemodynamic failure, to allow mechanical ventilation.

### Laboratory analysis

**HLA-A, HLA-B, HLA-C, HLA-DR, and HLA-DQ typing:** extraction of genomic deoxyribonucleic acid (DNA) from blood samples was performed using the QIAamp DNA mini extraction kit (Qiagen, Hilden, Germany). Gene typing of the HLA-A, HLA-B, HLA-C, HLA-DR, and HLA-DQ loci was performed by the PCR sequence-specific oligonucleotide (PCR-SSO) technique with the LABType™ SSO typing kits (One lambda Inc, USA). Interpretation was performed on the HLA FUSION™ software.

**Statistical analysis:** statistical analyses were performed using the SPSS statistical package (version 23.0, SPSS Inc, Chicago, IL, USA). First, a description analysis was run. Results were described using mean and standard deviation (SD) for continuous variables, numbers, and frequencies for categorical variables. Analytical analysis was then performed. Clinical and demographic characteristics of patients were compared between the deceased and non-deceased, intubated and non-intubated groups using univariate analysis. This comparison was made using the Student's t-test for continuous variables and Chi-square test or Fisher's exact test (when the theoretical number of patients was <5) for categorical variables. A p-value <0.05 was considered as significant. We studied then, the association between the different HLA alleles and the parameters of the COVID-19 prognosis, namely death and intubation using a multiple logistic regression model for multivariate analysis, with stepwise variable selection. This association study was performed by calculation of odds ratio and its adjustment to potential confounding factors: clinical, biological and demographic factors that had a p-value <0.3 in the univariate analysis. Thus, to investigate the association of HLA alleles and predisposition to death in the course of severe COVID-19, we performed a logistic regression analysis after adjustment to potential confounding factors: age, comorbidities, and blood type. To assess the association of HLA alleles with patients' progression to intubation, we used a logistic regression model after adjustment to sex, blood type, and history of cardiovascular disease.

**Ethical considerations:** the study was approved by the local ethics committee of Sahloul University Teaching Hospital on 13^th^ February 2021. All data and patients´ identities were processed with strict confidentiality.

## Results

**General characteristics of the COVID-19 patients hospitalized in ICU:** patients included in our study had a mean age of 64.5 ± 11.5 (34-83) years. The majority of patients were men (64.3%; (n=27)) with a sex ratio (M/F) of 1.8. The most common underlying diseases were obesity (61.9%; (n=26)), hypertension (26.2%; n=11), and diabetes (23.8%; (n=10)). Only one patient had asthma. Thirty-two patients died (76.2%). On admission, all patients had an oxygen saturation of less than 94% and were on mechanical ventilation. Eleven patients (26.2%) required intubation during hospitalization. The mean Acute Physiologic Assessment and Chronic Health Evaluation II (APACHE II) and Sequential Organ Failure Assessment (SOFA) scores levels were 17.38 ± 10.16 (6-45) and 7.79 ± 5 (2-20), respectively. Regarding the blood types, the most frequent blood type was group O positive (45.2%; (n=19)), followed by group A positive (35.7%; (n=15)) (Table 1). Comparing O-positive blood group carriers with non-O-positive patients, we found that the frequency of the O-positive blood group was significantly higher in surviving than in deceased COVID-19 patients (90% (n=9) versus 31.2% (n=10), p=0.01). Then, blood group O seems to be associated with reduced mortality risk with an OR of 0.114 (95% CI= 0.020-0.635). When comparing A-positive versus non-A-positive patients, we found that there was a tendency for A-positive blood group to be more frequent in deceased than in surviving COVID-19 patients (43.75% (n=14) versus 10% (n=1), p=0.061). Then, blood group A seems to be associated with increased mortality risk with an OR of 7.941 (95% CI= 0.020-0.635). No statistically significant association was found between blood type and risk of intubation (Table 1). All of the demographic and clinical characteristics of the patients studied are detailed in Table 1.

**Table 1:** clinical and epidemiological characteristics of COVID-19 patients hospitalized in the ICU

Characteristics	Total (N=42)	Death			Intubation		
		Deceased	Not deceased	P	Intubated	Not intubated	P
Age (years)	64.5±1.5	65.8±11.4	60.3±11.2	0.19	64.55±11.6	64.45±11.6	0.982
**Sex**							
Male	27/42	22/32	5/10	0.451	9/11	18/31	0.273
Female	15/42	10/32	5/10		2/11	13/31	
Smoking	10/42	7/32	3/10	0.678	2/11	8/31	1.000
Asthma	1/42	1/32	0	1.000	1/28	0	1.000
Comorbidities	32/42	26/32	6/10	0.213	9/11	23/31	1.000
Obesity	26/42	20/32	6/10	1.000	6/11	20/31	0.720
Hypertension	11/39	9/32	2/7	1.000	4/11	7/28	0.694
Diabetes	10/39	8/32	2/7	1.000	2/11	8/28	0.693
Cardiovascular disease	5/39	4/32	1/7	1.000	3/11	2/28	0.125
History of deep vein thrombosis	1/39	1/ 32	0	1.000	0	1/27	1.000
History of stroke	1/39	1/32	0	1.000	1/27	0	1.000
**Symptoms**							
Fever	16/35	13/31	3/4	0.312	3/11	13/24	0.138
Cough	16/35	13/31	3/4	0.312	4/11	12/24	0.452
Dyspnea	10/35	10/31	0	0.303	8/11	2/24	<0.0001
Headache	6/35	4/31	2/4	0.128	0	6/24	0.146
Thrombosis		12/32	0	0.04	6/11	6/31	0.049
**Blood group**				**0.03**			0.291
O positive	19/42 (45.2%)	10/32 (31.25%)	9/10 (90%)	0.01	3/11	16/31	0.291
A positive	15/42 (35.7%)	14/32 (43.75%)	1/10 (10%)	0.061	5/11	11/31	0.720
B positive	5/42 (11.9%)	5/32 (15.62%)	0	0.315	1/11	4/31	1.000
O negative	2/42 (4.76%)	2/32 (6.25%)	0	1.000	1/11	1/31	0.460
B negative	1/42 (2.4%)	1/32 (3.125%)	0	1.000	1/11	0	0.262

ICU: intensive care unit

**Study of HLA gene allele frequencies:** for each locus (A, B, C, DR, and DQ), the frequencies of the 84 HLA alleles are represented in Table 2, Table 3, and Table 4. The A*02 allele is the most frequent allele concerning locus A at 22.6% (n=19). The B*50 allele is the most frequent allele concerning the B locus at 14.3% (n=12). The C*06 allele is the most frequent allele concerning the C locus at 22.6% (n=19). For the locus DRB1, the DRB1*11 allele is the most frequent allele at 22.6% (n=19). Finally, the alleles DQA1*01 at 33.33% (n=28) and DQB1*03 (n=29; 34.5%) were the most frequent alleles for the DQ locus.

**Table 2:** frequencies of HLA class I alleles in deceased and not deceased COVID-19 patients hospitalized in ICU

Allele	HLA frequency in covid-19 patients n/N(%)	Death						Allele	HLA frequency in covid-19 patients n/N(%)	Death					
		Deceased n/N(%)	Not deceased n/N(%)	Unadjusted ORs (95% CI)	P	Adjusted ORs (95% CI)	P’			Deceased n/N(%)	Not deceased n/N(%)	Unadjusted ORs (95% CI)	P	Adjusted ORs (95% CI)	P’
A*01	12/84(14.3)	7/32(21.9)	4/10(40)	0.42(0.092-1.916)	0.410	0.215 (0.028-1.672)	0.142	B*42	2/84(2.4)	1/32(3.1)	1/10(10)	0.29(0.016-5.118)	0.424	0.044(0.0001-4.709)	0.191
A*02	19/84(22.6)	16/32(50)	3/10(30)	2.333(0.51-10.665)	0.305	1.467(0.235-9.145)	0.682	B*44	9/84(10.71)	5/32(15.6)	3/10(30)	0.432(0.083-2.262)	0.369	0.276(0.028-2.713)	0.270
A*03	8/84(9.52)	4/32(12.5)	4/10(40)	0.214(0.041-1.108)	0.075	0.151(0.017-1.299)	0.085	B*45	3/84(3.6)	2/32(6.2)	1/10(10)	0.6(0.049-7.408)	1	0.236(0.001-46.682)	0.592
A*11	5/84(5.95)	4/32(12.5)	1/10(10)	1.286(0.127-13.036)	1	0.606(0.039-9.459)	0.721	B*49	2/84(2.4)	2/32(6.2)	0	-	1	-	0.999
A*20	1/84(1.2)	1/32(3.1)	0	-	1	-	1	B*50	12/84(14.3)	8/32(25)	2/10(20)	1.333(0.233-7.626)	1	1.553(0.170-14.231)	0.697
A*23	6/84(7.14)	4/32(12.5)	1/10(10)	1.286(0.127-13.036)	1	13.38(0.19-927.286)	0.230	B*51	5/84(5.95)	2/32(6.2)	3/10(30)	0.156(0.022-1.115)	0.078	0.105(0.008-1.349)	0.084
A*24	3/84(3.6)	2/32(6.2)	1/10(10)	0.6(0.049-7.408)	1	0.127(0.001-13.381)	0.385	B*52	1/84(1.2)	1/32(3.1)	0	-	1	-	1
A*26	1/84(1.2)	1/32(3.1)	0	-	1	-	1	B*53	2/84(2.4)	2/32(6.2)	0	-	1	-	0.999
A*29	5/84(5.95)	4/32(21.5)	1/10(10)	1.286(0.127-13.036)	1	1.259(0.047-33.958)	0.891	B*55	2/84(2.4)	1/32(3.1)	0	-	1	-	1
A*30	9/84(10.71)	6/32(18.7)	2/10(20)	0.923(0.155-5.505)	1	0.973(0.118-8.041)	0.980	B*56	1/84(1.2)	1/32(3.1)	0	-	1	-	1
A*32	44/84(4.8)	3/32(9.3)	1/10(10)	0.931(0.086-10.095)	1	1.546(0.069-34.521)	0.783	B*57	2/84(2.4)	1/32(3.1)	1/10(10)	0.29(0.016-5.118)	0.424	0.265(0.005-12.892)	0.503
A*33	8/84(9.52)	6/32(18.7)	0	-	0.308	-	0.999	B*58	3/84(3.6)	2/32(6.2)	0	-	1	-	0.999
A*34	1/84(1.2)	1/32(3.1)	0	-	1	-	1	HLA-C (n(%))							
A*68	2/84(2.4)	1/32(3.1)	1/10(10)	0.29(0.016-5.118)	0.424	0.44(0.0001-4.709)	0.191	C*01	1/84(1.2)	1/32(3.1)	0	-	1	-	1
HLA-B (n(%))								C*02	3/84(3.6)	3/32(9.3)	0	-	1	-	0.999
B*07	4/84(4.8)	3/32(9.3)	1/10(10)	0.931(0.086-10.095)	1	0.256(0.006-11.219)	0.480	C*03	6/84(7.14)	3/32(9.3)	1/10(10)	0.828(0.075-9.074)	1	1.76(0.114-27.275)	0.686
B*08	1/84(1.2)	1/32(3.1)	0	-	1	-	1	C*04	16/84(19.04)	11/32(34.4)	2/10(20)	1.833(0.324-10.367)	0.692	1.813(0.242-13.59)	0.563
B*13	2/84(2.4)	2/32(6.2)	0	-	1	-	0.999	C*05	3/84(3.6)	2/32(6.2)	1/10(10)	0.533(0.043-6.655)	0.535	1.335(0.084-21.14)	0.838
B*14	3/84(3.6)	2/32(6.2)	1/10(10)	0.6(0.049-7.408)	1	1.716(0.08-36.643)	0.730	C*06	19/84(22.61)	10/32(31.2)	3/10(30)	0.909(0.188-4.39)	1	0.946(0.11-8.142)	0.959
B*15	3/84(3.6)	2/32(6.2)	0	-	1	-	0.999	C*07	13/84(15.5)	8/32(25)	2/10(20)	1.167(0.2-6.805)	1	1.885(0.076-46.751)	0.699
B*18	5/84(5.95)	1/32(3.1)	1/10(10)	0.29(0.016-5.118)	0.424	-	0.995	C*08	3/84(3.6)	2/32(6.2)	1/10(10)	0.533(0.043-6.655)	0.535	1.417(0.062-32.442)	0.827
B*35	10/84(11.9)	5/32(15.6)	3/10(30)	0.432(0.083-2.262)	0.369	0.293(0.035-2.459)	0.258	C*12	3/84(3.6)	3/32(9.3)	0	-	1	-	0.999
B*37	1/84(1.2)	1/32(3.1)	0	-	1	-	1	C*14	4/84(4.8)	3/32(9.3)	1/10(10)	0.828(0.075-9.074)	1	1.176(0.066-20.872)	0.912
B*38	1/84(1.2)	1/32(3.1)	0	-	1	-	1	C*15	1/84(1.2)	0	1/10(10)	-	0.220	-	1
B*40	5/84(5.95)	4/32(12.5)	1/10(10)	1.286(0.127-13.036)	1	1.461(0.131-16.347)	0.758	C*16	5/84(5.95)	4/32(12.5)	1/10(10)	1.143(0.111-11.722)	1	0.064(0.001-3.162)	0.167
B*41	5/84(5.95)	3/32(9.3)	2/10(20)	0.414(0.059-2.917)	0.577	0.69(0.037-12.797)	0.803	C*17	5/84(5.95)	2/32(6.2)	3/10(30)	0.133(0.018-0.978)	0.061	0.018(0.0001-0.814)	0.039

ICU: intensive care unit; P: value of Chi-square statistic analysis; P’: statistic value after adjustment to potential confounding factors by binary logistic regression

**Table 3:** frequencies of HLA class I alleles in intubated and not intubated COVID-19 patients hospitalized in ICU

Allele	HLA frequency in covid-19 patients n/N(%)	Intubation						Allele	HLA frequency in covid-19 patients n/N(%)	Intubation					
		Intubated n/N(%)	Not intubated n/N(%)	Unadjusted ORs (95% CI)	P	Adjusted ORs (95% CI)	P’			Intubated n(%) N=11	Not intubated n(%) N=31	Unadjusted ORs (95% CI)	P	Adjusted ORs (95% CI)	P’
A*01	12/84(14.3)	2/11(18.2)	9/31(29)	0.543(0.098-3.025)	0.696	0.593(0.078-4.526)	0.614	B*42	2/84(2.4)	1/11(9.1)	1/31(3.2)	3(0.171-52.527)	0.460	1.808(0.081-40.176)	0.708
A*02	19/84(22.6)	6/11(54.5)	13/31(40.6)	1.662(0.416-6.636)	0.504	1.015(0.203-5.071)	0.986	B*44	9/84(10.71)	3/11(27.2)	5/31(16.1)	1.95(0.38-10.013)	0.412	1.95(0.291-13.056)	0.491
A*03	8/84(9.52)	1/11(9.1)	7/31(22.5)	0.343(0.037-3.161)	0.657	0.243(0.015-3.857)	0.316	B*45	3/84(3.6)	0	3/31(9.7)	-	1	-	0.999
A*11	5/84(5.95)	1/11(9.1)	4/31(12.9)	0.675(0.067-6.789)	1	0.705(0.056-8.854)	0.787	B*49	2/84(2.4)	1/11(9.1)	1/31(3.2)	-	1	-	0.809
A*20	1/84(1.2)	0	1/31(3.2)	-	1	-	1	B*50	12/84(14.3)	2/11(18.2)	8/31(25.8)	0.639(0.113-3.606)	1	0.407(0.056-2.971)	0.375
A*23	6/84(7.14)	0	5/31(16.1)	-	0.303	-	0.999	B*51	5/84(5.95)	1/11(9.1)	4/31(12.9)	0.675(0.067-6.789)	1	0.63(0.23-17.739)	0.787
A*24	3/84(3.6)	2/11(18.2)	1/31(3.2)	6.667(0.54-82.31)	0.163	3.775(0.241-59.1)	0.344	B*52	1/84(1.2)	1/11(9.1)	0	-	0.262	-	1
A*26	1/84(1.2)	0	1/31(3.2)	-	1	-	1	B*53	2/84(2.4)	0	2/31(6.4)	-	1	-	0.999
A*29	5/84(5.95)	2/11(18.2)	3/31(9.7)	2.074(0.298-14.439)	0.593	14.5(0.491-428.7)	0.122	B*55	2/84(2.4)	0	1/31(3.2)	-	1	-	1
A*30	9/84(10.71)	1/11(9.1)	7/31(22.5)	0.343(0.037-3.161)	0.657	0.476(0.044-5.094)	0.539	B*56	1/84(1.2)	1/11(9.1)	0	-	0.262	-	1
A*32	44/84(4.8)	2/11(18.2)	2/31(6.4)	3.222(0.395-26.255)	0.277	4.017(0.352-45.873)	0.263	B*57	2/84(2.4)	1/11(9.1)	1/31(3.2)	3(0.171-52.527)	0.460	6.345(0.117-345.3)	0.365
A*33	8/84(9.52)	2/11(18.2)	4/31(12.9)	1.5(0.234-9.611)	0.644	0.504(0.048-5.285)	0.568	B*58	3/84(3.6)	2/11(18.2)	0	-	0.064	-	0.999
A*34	1/84(1.2)	0	1/31(3.2)	-	1	-	1	HLA-C (n(%))							
A*68	2/84(2.4)	1/11(9.1)	1/31(3.2)	3(0.171-52.527)	0.460	47.61(0.658-3444.6)	0.077	C*01	1/84(1.2)	1/11(9.1)	0	-	0.262	-	1
HLA-B (n(%))								C*02	3/84(3.6)	2/11(18.2)	1/31(3.2)	-	0.17	-	0.456
B*07	4/84(4.8)	1/11(9.1)	3/31(9.7)	0.933(0.087-10.040)	1	0.643(0.51-8.154)	0.733	C*03	6/84(7.14)	2/11(18.2)	2/31(6.4)	3.111(0.381-25.379)	0.288	10.89(0.446-267.04)	0.144
B*08	1/84(1.2)	0	1/31(3.2)	-	1	-	1	C*04	16/84(19.04)	1/11(9.1)	12/31(38.7)	0.150(0.017-1.329)	0.127	0.06(0.003-1.067)	0.055
B*13	2/84(2.4)	0	2/31(6.4)	-	1	-	0.999	C*05	3/84(3.6)	0	3(9.7)	-	0.551	-	0.999
B*14	3/84(3.6)	0	3/31(9.7)	-	0.554	-	0.999	C*06	19/84(22.61)	3/11(27.2)	10/31(32.2)	0.75(0.163-3.459)	1	0.838(0.147-4.765)	0.842
B*15	3/84(3.6)	1/11(9.1)	1/31(3.2)	3(0.171-52.527)	0.460	4.435(0.194-101.285)	0.351	C*07	13/84(15.5)	3/11(27.2)	7/31(22.5)	1.232(0.255-5.944)	1	1.453(0.236-8.94)	0.687
B*18	5/84(5.95)	0	2/31(6.4)	-	1	-	0.999	C*08	3/84(3.6)	0	3/31(9.7)	-	0.551	-	0.999
B*35	10/84(11.9)	1/11(9.1)	7/31(22.5)	0.343(0.037-3.161)	0.657	0.306(0.023-4.056)	0.369	C*12	3/84(3.6)	1/11(9.1)	2/31(6.4)	1.4(0.114-17.170)	1	0.741(0.039-13.927)	0.841
B*37	1/84(1.2)	1/11(9.1)	0	-	0.262	-	1	C*14	4/84(4.8)	2/11(18.2)	2/31(6.4)	3.111(0.381-25.379)	0.288	3.036(0.213-43.203)	0.412
B*38	1/84(1.2)	0	1/31(3.2)	-	1	-	1	C*15	1/84(1.2)	0	1/31(3.2)	-	1	-	1
B*40	5/84(5.95)	1/11(9.1)	4/31(12.9)	0.675(0.067-6.789)	1	1.003(0.073-13.868)	0.998	C*16	5/84(5.95)	2/11(18.2)	3/31(9.7)	2(0.287-13.942)	0.598	1.885(0.193-18.430)	0.586
B*41	5/84(5.95)	1/11(9.1)	4/31(12.9)	0.675(0.067-6.789)	1	3.665(0.207-64.965)	0.376	C*17	5/84(5.95)	1/11(9.1)	4/31(12.9)	0.65(0.065-6.545)	1	1.128(0.072-17.761)	0.932

ICU: intensive care unit; P: value of Chi-square statistic analysis; P’: statistic value after adjustment to potential confounding factors by binary logistic regression; HLA: human leukocyte antigen

**Table 4:** frequencies of HLA class II alleles (HLA-DRB1, and HLA-DQB1) in COVID-19 patients hospitalized in ICU

Allele	HLA frequency in covid-19 patients n/N(%)	Death						Intubation					
		Deceased n/N(%)	Not deceased n/N(%)	Unadjusted ORs (95% CI)	P	Adjusted ORs (95% CI)	P’	Intubated n/N(%)	Not intubated n/N(%)	Unadjusted ORs (95% CI)	P	Adjusted ORs (95% CI)	P’
**HLA-DRB1**													
DRB1*01	5/84(5.95)	2/32(6.2)	3/10(30)	0.156(0.022-1.115°	0.078	0.183(0.014-2.477)	0.201	1/11(9.1)	4/31(12.9)	0.675(0.067-6.789)	1	2.241(0.102-49.247)	0.609
DRB1*03	8/84(9.52)	6/32(18.7)	1/10(10)	2.077(0.219-19.673)	1	5.228(0.319-85.606)	0.246	3/11(27.2)	4/31(12.9)	2.531(0.466-13.747)	0.353	1.351(0.173-10.563)	0.774
DRB1*04	11/84(13.1)	6/32(18.7)	3/10(30)	0.538(0.107-2.715)	0.660	0.543(0.074-3.982)	0.548	2/11(18.2)	7/31(22.5)	0.762(0.133-4.377)	1	0.726(0.095-5.557)	0.758
DRB1*07	16/84(19.04)	12/32(37.5)	2/10(20)	2.4(0.435-13.227)	0.451	2.648(0.332-21.149)	0.358	5/11(45.4)	9/31(29)	2.037(0.493-8.408)	0.459	1.877(0.349-10.095)	0.463
DRB1*08	2/84(2.4)	2/32(6.2)	0	-	1	-	0.999	0	2/31(6.4)	-	1	-	0.999
DRB1*10	1/84(1.2)	1/32(3.1)	0	-	1	-	1	1/11(9.1)	0	-	0.262	-	1
DRB1*11	19/84(22.61)	10/32(31.2)	4/10(40)	0.682(0.157-2.964)	0.707	1.345(0.204-8.879)	0.758	2/11(18.2)	11/31(35.4)	0.352(0.065-1.915)	0.283	0.435(0.065-2.905)	0.390
DRB1*13	8/84(9.52)	5/32(15.6)	3/10(30)	0.432(0.083-2.262)	0.369	0.104(0.009-1.214)	0.071	0	8/31(25.8)	-	0.086	-	0.999
DRB1*14	4/84(4.8)	4/32(12.5)	0	-	0.557	-	0.998	2/11(18.2)	2/31(6.4)	3.222(0.395-26.255)	0.277	4.669(0.384-56.729)	0.227
DRB1*15	9/84(10.71)	6/32(18.7)	1/10(10)	2.077(0.219-19.678)	1	0.403(0.024-6.785)	0.526	3/11(27.2)	4/31(12.9)	2.531(0.466-13.747)	0.353	5.411(0.583-50.252)	0.138
DRB1*16	1/84(1.2)	0	1/10(10)	-	0.238	-	0.998	0	1/31(3.2)	-	1	-	1
HLA-DQA1													
HLA-DQA1*01	28/84(33.33)	15/32(46.9)	7/10(70)	0.378(0.083-1.73)	0.284	0.152(0.017-1.35)	0.091	7/11(63.6)	15/31(48.3)	1.867(0.453-7.693)	0.384	7.336(0.726-74.124)	0.091
HLA-DQA1*02	15/84(17.9)	11/32(34.4)	2/10(20)	2.095(0.378-11.615)	0.466	2.634(0.328-21.14)	0.362	4/11(36.3)	9/31(29)	1.397(0.327-5.971)	0.713	1.321(0.221-7.9)	0.760
HLA-DQA1*03	9/84(10.71)	4/32(12.5)	3/10(30)	0.333(0.06-1.844)	0.328	0.527(0.071-3.929)	0.532	0	7/31(22.5)	-	0.161	-	0.999
HLA-DQA1*04	1/84(1.2)	1/32(3.1)	0	-	1	-	1	1/11(9.1)	0	-	0.262	-	1
HLA-DQA1*05	31/84(36.9)	18/32(56.2)	5/10(50)	1.286(0.31-5.334)	1	1.61(0.267-9.709)	0.604	6/11(54.5)	17/31(54.8)	0.988(0.248-3.935)	1	0.531(0.086-3.302)	0.498
**HLA-DQB1**													
HLA-DQB1*02	24/84(28.6)	16/32(50)	4/10(40)	1.5(0.355-6.347)	0.723	2.264(0.361-14.222)	0.383	7/11(63.6)	13/31(41.9)	2.423(0.585-10.03)	0.216	3.026(0.464-19.734)	0.247
HLA-DQB1*03	29/84(34.5)	17/32(53.1)	6/10(60)	0.756(0.178-3.199)	1	1.117(0.188-6.649)	0.903	3/11(27.2)	20/31(64.5)	0.206(0.045-0.94)	0.043	0.151(0.023-0.976)	0.047
HLA-DQB1*04	3/84(3.6)	2/32(6.2)	1/10(10)	0.6(0.049-7.408)	1	0.127(0.001-13.381)	0.385	1/11(9.1)	2/31(6.4)	1.45(0.118-17.767)	1	0.774(0.049-12.231)	0.856
HLA-DQB1*05	13/84(15.5)	6/32(18.7)	4/10(40)	0.346(0.074-1.624)	0.213	0.504(0.067-3.799)	0.5066	3/11(27.2)	7/31(22.5)	1.286(0.267-6.189)	1	2.235(0.316-15.806)	0.420
HLA-DQB1*06	15/84(17.85)	9/32(28.1)	4/10(40)	0.587(0.133-2.582)	0.697	0.066(0.005-0.821)	0.035	4/11(36.3)	9/31(29)	1.397(0.327-5.971)	0.713	1.926(0.314-11.817)	0.479

ICU: intensive care unit; P: value of Chi-square statistic analysis; P’: statistic value after adjustment to potential confounding factors by binary logistic regression; HLA: human leukocyte antigen

**Association between HLA alleles and death:** we found that the HLA DQB1*06 allele was significantly associated with protection against mortality aOR: 0.066, 95% CI 0.005-0.821; p = 0.035.

**Association between HLA alleles and intubation:** it was found that the HLA DQB1*03 allele was significantly associated with protection against intubation aOR: 0.151, 95% CI 0.023-0.976; p = 0.047.

## Discussion

In addition to non-genetic factors such as age, gender, and comorbidities, genetic factors may influence the evolution and prognosis of COVID-19 [6]. Thus, we were interested in analyzing HLA genes´ role in COVID-19 outcomes in critically ill patients. We found that the HLA DQB1*06 allele was significantly associated with protection against mortality and the HLA DQB1*03 allele was significantly associated with protection against intubation. Regarding blood types, we found that the O-positive blood group was significantly more frequent in surviving patients than in deceased patients. On the contrary, the A-positive group tended to be more frequent in the deceased than in the survivors.

Our results, concerning blood types, were in line with the data in the literature. According to a recent literature review [6], group O was protective against SARS-CoV-2 infection and severity. Group A was, on the contrary, a predisposing factor to severity and SARS-CoV-2 infection. A Tunisian study also confirms these data concerning the risk of SARS-CoV-2 infection [15]. A Chinese study, like the present study, found the same association of blood groups O and A with the risk of mortality by SARS-CoV-2 [16]. The protective role of blood group O may be explained by the presence of natural anti-A and anti-B antibodies that inhibit the attachment of the virus to its cellular receptor [16].

Concerning HLA alleles in these critically ill COVID-19 patients, we found a tendency of the HLA DQB1*06 allele to be protective against death and the HLA DQB1*03 to be protective against progression to intubation. This protective role of HLA DQB1*06 and HLA DQB1*03 alleles has been verified in many other infectious diseases [17-23]. Indeed, HLA DQB1*03 was found to confer an immune protection in Human Immunodeficiency Virus (HIV) infection [17] and was also associated with Hepatitis C Virus (HCV) spontaneous clearance [18]. It was also found in a recent study that HLA DQB1*03 was protective against the severity of the Middle East Respiratory Syndrome Coronavirus (MERS-CoV) infection and associated with the development of a minor to moderate form of this infection [19]. This finding is in agreement with our results since MERS-CoV belongs to the same coronavirus family as SARS-CoV-2 [19]. HLA DQB1*06 allele, and more precisely HLA DQB1*06: 04 and HLA DQB1*06: 03 were protective against hepatitis B virus (HBV) infection. It was associated with spontaneous clearance of HBV and with a better evolution of the infection [20]. Furthermore, HLA DQB1*06: 03 and HLA DQB1*06: 09 were associated with resistance to HIV infection [21]. In fact, HLA DQB1*06 was found to provide robust and poly-functional mucosal CD4+ T cell responses against HIV [22]. A Chinese study also found that HLA DQB1*06 had protective effect on the evolution of HIV infection and T-cell targeting of specific HIV Nef epitopes. This study even proposes the consideration of the presence of HLA DQB1*06 in the production of the anti-HIV vaccine, given the role of this allele in the promotion of specific anti-HIV lymphocyte immune response [24]. Regarding Epstein Barr Virus (EBV) infection, it was found that people homozygous for HLA DQB1*06 were more likely to be EBV seronegative than other DQB1 combinations [23].

Like our study, and during this COVID-19 pandemic, several authors have also examined the association of HLA alleles with severity, prognosis, and mortality of COVID-19. For example, a multicenter study in Europe found that carriers of the HLA C*04: 01 allele had twice the risk of being intubated when they had COVID-19 [9]. In a Chinese study, HLA-C*14: 02, B*51: 01, and A*11: 01 alleles were associated with disease severity. For HLA class II alleles, DRB1*14: 04, DRB1*01: 01, and DQA1*01: 01 were predisposing for severity risk, whereas DPB1*03: 01 and DRB1*12: 01 were rather protective in this Chinese study [25]. An American study showed that HLA B*53 was associated with a worse prognosis in black patients with COVID-19 [10]. Concerning mortality, an Egyptian study found that the HLA B*15 allele was protective against mortality [11]. While, according to Lorente L *et al*. the HLA A*11, HLA C*01, and HLA DQB1*04 alleles were associated with mortality in patients hospitalized in ICU for COVID-19 [8]. Thus, regarding the association between HLA alleles and disease severity, the published articles report very varied results. This discrepancy of results is probably due to the lack of common criteria for the classification of disease severity, differences in the choice of groups of subjects compared, and especially the ethnic differences of the groups studied. Despite this variability in results, the search for an association between HLA system and the course of COVID-19 remains fully justified, given the crucial role of this system in the initiation of the antiviral immune response. Indeed, the HLA system codes for proteins on the cell surface. They present foreign peptides, including viral peptides, to immune T cells. This allows activation of the adaptive cellular or humoral antiviral immune response. However, the HLA system is highly polymorphic. The high polymorphism of HLA molecules affects the peptide binding groove. This allows presenting of different repertoires of peptides. Thus, different genetic polymorphisms of HLA have been associated with the predisposition and/or outcome of different infectious diseases [6]. These findings prompted the authors to investigate a possible relationship between HLA polymorphism and COVID-19.

Our study has some limitations, mainly the small sample size. Despite that, we were able to obtain preliminary results regarding the association between HLA alleles and COVID-19 prognosis in critically ill patients. The particular strength of this association study was the consideration of patients' blood types in addition to other clinical and demographic characteristics. Besides, we had the opportunity to perform HLA typing of several loci, namely A, B, C, DR, DQA1, and DQB1. Our findings may be a first step in optimizing personalized treatment and better managing of COVID-19 patients. They may help also to contribute to understanding the host-pathogen interaction.

## Conclusion

In this study, it was found that there are 2 protective HLA alleles against COVID-19 severity and mortality in critically ill patients. To our knowledge, this association hasn´t been studied before in Tunisia. Thus, there is a need to increase the sample size and study this association in other regions. Our results could be a first step to identifying individuals genetically susceptible or on the contrary protected against severe forms of COVID-19 in Tunisia and consequently to adopt a personalized treatment.

### What is known about this topic

- *Age, gender, and comorbidities influence the outcome of COVID-19;*
- *HLA system influences the outcome of several infectious diseases;*
- *Association of HLA polymorphism with the outcome of COVID-19 is still controversial*.

### What this study adds

- *HLA polymorphism may influence the outcome of COVID-19;*
- HLA DQB1*06 allele was significantly associated with protection against mortality;
- HLA DQB1*03 allele was significantly associated with protection against intubation.
